# CARM1 promotes gastric cancer progression by regulating TFE3 mediated autophagy enhancement through the cytoplasmic AMPK-mTOR and nuclear AMPK-CARM1-TFE3 signaling pathways

**DOI:** 10.1186/s12935-022-02522-0

**Published:** 2022-03-04

**Authors:** Suzhen Yang, Jing Zhang, Di Chen, Jiayi Cao, Ying Zheng, Yuying Han, Yirong Jin, Shuhui Wang, Ting Wang, Lin Ma, Tingting Luo, Yan Wang, Wen Qin, Lei Dong

**Affiliations:** 1 Department of Digestive Disease and Gastrointestinal Motility Research Room, The Second Affiliated Hospital of Xi’an Jiaotong University, Xi’an, People’s Republic of China; 2 State Key Laboratory of Military Stomatology and National Clinical Research Center for Oral Diseases and Shaanxi Clinical Research Center for Oral Diseases, Department of Orthodontics, School of Stomatology, Fourth Military Medical University, Xi’an, 710032 People’s Republic of China; 3 Department of Kidney Transplantation, Nephropathy Hospital, The First Affiliated Hospital of Xi’an Jiaotong University, Xi’an, 710061 People’s Republic of China; 4 State Key Laboratory of Cancer Biology, National Clinical Research Center for Digestive Diseases and Xijing Hospital of Digestive Diseases, Air Force Military Medical University, Xi’an, 710032 People’s Republic of China; 5 Faculty of Life Science, Northwest University, 229 Taibai North Road, Xi’an, 710069 Shaanxi Province People’s Republic of China; 6 Department of Infectious Diseases, Shenzhen Shekou People’s Hospital, Shenzhen, 518067 People’s Republic of China; 7 Shaanxi Provincial People’s Hospital, Xi’an, 710043 Shaanxi People’s Republic of China

**Keywords:** Autophagy, CARM1, TFE3, AMPK, Gastric cancer

## Abstract

**Background:**

The role of CARM1 in tumors is inconsistent. It acts as an oncogene in most cancers but it inhibits the progression of liver and pancreatic cancers. CARM1 has recently been reported to regulate autophagy, but this function is also context-dependent. However, the effect of CARM1 on gastric cancer (GC) has not been studied. We aimed to explore whether CARM1 was involved in the progression of GC by regulating autophagy.

**Methods:**

The clinical values of CARM1 and autophagy in GC were evaluated by immunohistochemistry and qRT–PCR. Transmission electron microscopy, immunofluorescence and western blotting were employed to identify autophagy. The role of CARM1 in GC was investigated by CCK-8, colony formation and flow cytometry assays in vitro and a xenograft model in vivo. Immunoprecipitation assays were performed to determine the interaction of CARM1 and TFE3.

**Results:**

CARM1 was upregulated in clinical GC tissues and cell lines, and higher CARM1 expression predicted worse prognosis. CARM1 enhanced GC cell proliferation, facilitated G1-S transition and inhibited ER stress-induced apoptosis by regulating autophagy. Importantly, treatment with a CARM1 inhibitor rescued the tumor-promoting effects of CARM1 both in vitro and in vivo. Furthermore, we demonstrated that CARM1 promoted TFE3 nuclear translocation to induce autophagy through the cytoplasmic AMPK-mTOR and nuclear AMPK-CARM1-TFE3 signaling pathways.

**Conclusion:**

CARM1 promoted GC cell proliferation, accelerated G1-S transition and reduced ER stress-induced apoptosis by regulating autophagy. Mechanistically, CARM1 triggered autophagy by facilitating TFE3 nuclear translocation through the AMPK-mTOR and AMPK-CARM1-TFE3 signaling pathways.

**Supplementary Information:**

The online version contains supplementary material available at 10.1186/s12935-022-02522-0.

## Introduction

Gastric cancer(GC) is the fifth most commonly diagnosed malignant tumor and the fourth leading cause of cancer death globally [1], causing a severe economic burden. Multidisciplinary treatment has become the mainstay for GC patients, especially with the development of immunotherapy and targeted therapy [2]. However, the number of patients benefiting from the new treatment is limited due to the heterogeneity of the patient population. Therefore, it is critical to investigate the fundamental molecular mechanisms of GC pathogenesis and identify new diagnostic biomarkers and therapeutic targets.

Autophagy is a self-eating process that recycles wastes of cells and maintains homeostasis by clearing longevity proteins or damaged organelles. These cellular contents and organelles are sequestered in double-membrane structures called autophagosomes, which are then fused with lysosomes and degraded [3]. Autophagy is activated when cells encounter environmental stress, such as malnutrition, hypoxia, pathogen infection, and oxidative stress, and cells either adapt or die depending on the intensity of the stimulus and the response of the host [4]. Autophagy is reported to exhibit crucial but conflicting functions in the progression of various tumors, including pancreatic adenocarcinoma, myeloid leukemia, gastric carcinoma, squamous cell carcinoma, sarcoma, and multiple myeloma [5–8]. More importantly, there have been several clinical trials targeting autophagy in malignant glioma, non-small cell lung cancer, colon cancer, melanoma, pancreatic cancer and melanoma, implicating promising prospects for targeting autophagy therapy [9].

Arginine methylation, which is regulated by the protein arginine methyltransferase (PRMT) family, is one of the most vital epigenetic modifications in autophagy [10]. Coactivator-associated arginine methyltransferase-1 (CARM1) plays a special role because it is the only enzyme capable of methylating arginine with proline sequences [11]. CARM1 has two main domains, which are required for the interaction with chromatin remodeling proteins and proteins possessing RNA binding activity, and the resultant protein complex plays essential roles in chromatin remodeling, gene transcription, DNA packing, pre-mRNA splicing, and regulation of mRNA stability [12, 13]. The role of CARM1 in cancers is paradoxical. It acts as an oncogene in lung, prostate, colorectal, ovarian, breast cancer, osteosarcoma, myeloid leukemia and multiple myeloma [14–23]. However, CARM1 inhibits the progression of liver and pancreatic cancers [19, 20]. Previous studies have mainly focused on the role of CARM1 as a coactivator in regulating tumor-related gene expression [24, 25]. Recent studies have shown that CARM1 is an important regulator of autophagy which functions mainly through two dependent pathways. In the nucleus, AMPK is activated under glucose starvation and subsequently phosphorylates and activates FOXO3a. This leads to a decrease in SKP2 expression and resulting in the stabilization of CARM1 in the nucleus, which acts as a coactivator of TFEB to promote autophagy-related gene transcription [26]. In the cytoplasm, C9orf72 mediates CARM1 lysosomal degradation by interacting with the PH-like domain and modulates lipid metabolism [27]. However, the role of CARM1 in GC has not been reported previously, and it remains unclear whether CARM1 could affect the progression of GC by regulating autophagy.

In this study, we illustrate the effect of CARM1 on GC and the mechanism by which CARM1 regulates autophagy. CARM1 and autophagy markers were upregulated in GC tissues, and higher CARM1 expression predicted worse prognosis. Knockdown of CARM1 interfered with the orderly progression of autophagy and subsequent ER stress-induced apoptosis and reduced cell proliferation by promoting G1 phase cell cycle arrest, which could be partially reversed by the autophagy agonist rapamycin. Overexpression of CARM1 exhibited the opposite results. More importantly, treatment with a CARM1 inhibitor rescued the tumor-promoting effects of CARM1 both in vitro and in vivo. Furthermore, we demonstrated that CARM1 facilitated TFE3 nuclear translocation to induce autophagy through the cytoplasmic AMPK-mTOR and nuclear AMPK-CARM1-TFE3 signaling pathways. Thus, our findings suggest that CARM1, either by itself or in combination with other signaling molecules, may serve as a novel therapeutic target for the treatment of GC.

## Methods

### Tissue microarray and clinical samples

Tissue microarrays (G6038-3), which comprise 48-paired GC tissues and matched adjacent non-neoplastic tissues were obtained from Servicebio Biotech (Wuhan, China). They contained clinical information such as sex, age, tumor location and size, metastasis, vessel carcinoma embolus and the overall survival of patients. After the immunohistochemical staining, immunostaining intensity was scored by two experienced pathologists, who were blinded of clinical information. The percentage of positive cells and the staining intensity were evaluated semi-quantitatively, the percentage of positive cells was divided into 5 grades and scores were assigned as follows: the number of positive cells < 5% was 0, 5–25% was 1, 26–50% was 2, 51–75% was 3, 76–100% was 4 points. The staining intensity was graded in 4 grades: negative was 0, weak was 1, moderate was 2, and strong was 3 points. The total scores, ranging between 0 and 12, were calculated by multiplying the positivity score (0–4) by the intensity score (0–3). Tissues with a total score < 7 were classified as low expression, while those with a score ≥ 7 were classified as high expression. Thirty-three pairs of clinical tissues were collected from the Second Affiliated Hospital of Xi’an Jiaotong University with informed consent from patients.

### Immunohistochemistry (IHC)

Tissue microarrays and paraffin sections were soaked in xylene twice for deparaffinization. The slides were rehydrated with a series of ethanol solutions (100%, 95%, 85% and 75%). Slides were evenly spaced in citrate buffer and microwaved for perform antigen retrieval. Subsequently, 3% hydrogen peroxide was added to remove endogenous peroxidase, and then the slides were blocked with goat serum for 30 min at room temperature (RT). Primary antibodies were added at appropriate concentrations and the slides were incubated at 4 °C overnight. The next day, biotinylated secondary antibodies were added and the slides were incubated at RT for 1 h. To visualize antibody binding, fresh DAB staining solution was added, and the staining process was monitored under a microscope. When the staining was complete, slides were counterstained in hematoxylin bath for 30–60 s, dehydrated in graded ethanol (75%, 85%, 95% and 100%) transferred to dimethylbenzene, and sealed in neutral resin.

### RNA extraction and qRT–PCR

Total RNA was extracted by TRIzol (Invitrogen, Thermo, Waltham, MA, USA). The messenger RNA was reverse-transcribed with the Transcriptor First-Strand cDNA Synthesis Kit (Roche, Basel, Switzerland). Subsequently, FastStart Universal SYBR Green Master (Roche) was used to conduct qRT**–**PCR analysis following the manufacturer’s instructions. All primer sequences are listed in Additional file 4: Table S2.

### Cell culture

The human GC cell lines HGC27, BGC823, MKN45, SNU-1 and the normal gastric epithelial cell line GES1 were purchased from GeneChem (Shanghai, China), identified by STR profiling and tested free of mycoplasma contamination. All cell lines were cultured in high glucose DMEM (HyClone, Logan, Utah, USA) supplemented with 10% fetal bovine serum (Gemini, Calabasas, CA, USA) and 1% penicillin–streptomycin (Gibco, Grand Island, NY, USA) in a humidified incubator in 5% CO_2_ at 37 °C.

### Western blotting

Total protein was extracted using cold RIPA buffer (Beyotime, Shanghai, China). Cytoplasmic and nuclear extracts were separated by NE-PER Nuclear and Cytoplasmic Extraction Reagents (Thermo Scientific™, MA, USA). The protein concentration of each cell lysate was determined using a BCA Protein Assay Kit (TIANGEN, Beijing, China). The equal amount of protein was separated by SDS-PAGE gel and transferred to PVDF membranes. The membrane was incubated overnight at 4 °C in a primary antibody solution. The antibodies used in this study are shown in Additional file 5: Table S3. The next day, the membrane was incubated with horseradish peroxidase-conjugated secondary antibody and visualized with enhanced chemiluminescence reagents.

### Lentivirus and siRNA transfection

The overexpression lentivirus and control lentivirus were obtained from Hanbio Biotechnology (Shanghai, China). The overexpression lentivirus was HBLV-h-CARM1-3xflag-ZsGreen-PURO, and the control vector was HBLV-ZsGreen-PURO. Transduction was performed according to the manufacturer’s protocol. Transduced cells were cultured in medium containing 2.5 μg/mL puromycin to select for cells stably expressing the transduced construct. HGC27 and BGC823 CARM1-overexpressing cells were transfected with specific siRNAs (GenePharma, Shanghai, China) targeting CARM1 and TFE3, respectively. Lipofectamine 2000 reagent (Invitrogen, Carlsbad, CA) was used for transfection in accordance with the manufacturer’s instructions. The interference sequences used in this study are shown in Additional file 4: Table S2.

### Transmission electron microscopy

Cells were harvested and fixed at 4 °C for 2–4 h with electron microscope fixation solution (Servicebio, China) and further fixed with 1% osmium in·0.1 M phosphate buffer for 2 h. Cells were dehydrated in graded ethanol (50%, 70%, 80%, 90%, 95% and 100%) and 100% acetone twice. Cells were embedded and cut into slices of 60–80 nm thickness with an ultra-thin slicing machine. Then, the sections were double-stained with 2% uranium acetate-lead citrate. Finally, photographs of eight random fields were captured for each sample under a transmission electron microscope (HITACHI, Tokyo, Japan).

### Immunofluorescence staining

Cells were cultured on small cover glass. 4% paraformaldehyde was added into the hole for cell fixation for 30 min at RT, cells were permeabilized with 0.1% Triton 100 for 15 min. Subsequently, cells were blocked with 5% goat serum and incubated without washing with a sufficient amount of primary antibodies at appropriate concentration overnight in a moist container at 4 °C. The following day, the cells were incubated with fluorescence-labeled secondary antibody for 1 h and DAPI for 1 min in the dark. Finally, the coverslip was sealed with anti-fading buffer (BD Biosciences, NJ, USA). Photographs of five random fields were captured under a confocal microscope (Nikon C2, Tokyo, Japan).

### CCK-8 assay

Cell suspensions were added to 96-well plates at 3 × 10^3^ cells per well in 100 μL DMEM and incubated for 1–4 days. Ten microliters of CCK-8 reagent (7 Sea Pharmatech Co., Ltd, Shanghai, China) was added to each well and the plates were incubated in a cell incubator for 1 h. The optical absorbance was read at 450 nm on a microplate spectrophotometer (Thermo, Waltham, MA, USA).

### Colony formation assay

Cells in the exponential growth phase were plated at a density of 1 × 10^3^ cells per well in 6-well plates and cultured for 10–14 days until visible colonies were formed. Cells were fed with fresh culture medium in a timely manner according to the pH change. When the colonies were ready for analysis, the cells were fixed with 4% paraformaldehyde for 20 min and stained with crystal violet dye for 10 min. Finally, the number of colonies containing more than 50 cells was counted.

### Flow cytometry

A total of 2 × 10^5^ cells were plated in 6-well plates, grown in serum-free medium for 24 h to synchronize the cell cycle, and cultured for another 24 h. The adherent cells were digested and fixed with 70% ice-cold ethanol at 4 °C overnight. The next day, the fixed cells were washed and resuspended in 500 μL PI/RNase Staining Buffer (BD Biosciences, Franklin Lakes, NJ, USA). After incubating for 15 min in the dark at RT, cell cycle was analyzed by flow cytometry (BD Biosciences, Franklin Lakes, NJ, USA).

A total of 1.5 × 10^5^ cells were seeded in each well of 6-well plates and cultured to a confluence of 80%-90%. Afterwards, the proportion of apoptotic cells was determined by staining with a PE Annexin V/7-amino-actinomycin (7-AAD) Detection Kit (BD Biosciences, NJ, USA) following the manufacturer’s instructions and analyzed by flow cytometry.

### In vivo xenograft tumor model

A subcutaneous xenograft model was established using 5 to 6-week-old male BALB/c nude mice (Xi’an Jiaotong University Medical Laboratory Animal Center, Xi’an, China). The experiments were approved by the animal ethics committee of Xi'an Jiaotong University. One million (1.0 × 10^6^) tumor cells were injected subcutaneously into the left groin of nude mice where blood flow was abundant. The volume of subcutaneous tumor was evaluated every 3 days. To test the efficacy of drug treatment, nude mice injected with CARM1-overexpressing cells were randomly divided into four groups on day 6 when tumor volumes reached 50 mm^3^. The CARM1 inhibitor EZM2302 was administered twice a day at 100 mg/kg i.p. HCQ was administered once a day at 50 mg/kg i.p. The combination group received both EZM2302 and HCQ treatments and the control group was intraperitoneally injected with PBS. When the subcutaneous tumor showed ulceration and necrosis, the mice were anesthetized with 3% sodium pentobarbital and euthanized with a high level of carbon dioxide. Then, the transplanted tumors were removed, and the weight and volume were measured.

### Immunoprecipitation assay

The cells were harvested and incubated with an appropriate amount of IP lysis buffer (containing protease inhibitor) for 30 min at 4 °C. After centrifugation, 6 μg CARM1 antibody or control IgG and 40 μL Protein A/G Mix Magnetic Beads (Merck Millipore, Germany) were added to the supernatant and incubated at 4 °C overnight. After the immunoprecipitation reaction, the protein was carefully eluted from the protein A/G-beads and denatured for western blotting analysis.

### Statistical analysis

We used GraphPad Prism 6 (San Diego, CA, USA) and SPSS 20.0 (Chicago, IL, USA) to perform statistical analysis. When comparing the differences in measurement data between the two groups, Student’s *t* test was used. When comparing the differences between more than two groups, ANOVA was applied, in which *p* values were adjusted for multiple comparisons. Before Student’s *t* test and ANOVA, a variance homogeneity test and normality analysis were carried out. Overall survival curves were analyzed by the Kaplan–Meier method and log-rank tests. The chi-squared test was utilized to estimate the difference in CARM1 levels between GC tissues and adjacent non-tumor tissues. The hazard ratio in clinical samples was determined by a Cox proportional hazards model for univariate and multivariate analyses. All values demonstrate means ± SD. All statistical tests were two-sided, and *P* < 0.05 was considered statistically significant.

## Results

### CARM1 and autophagy markers are upregulated in human GC tissues and higher CARM1 expression demonstrates poor prognosis

**Fig. 1:**
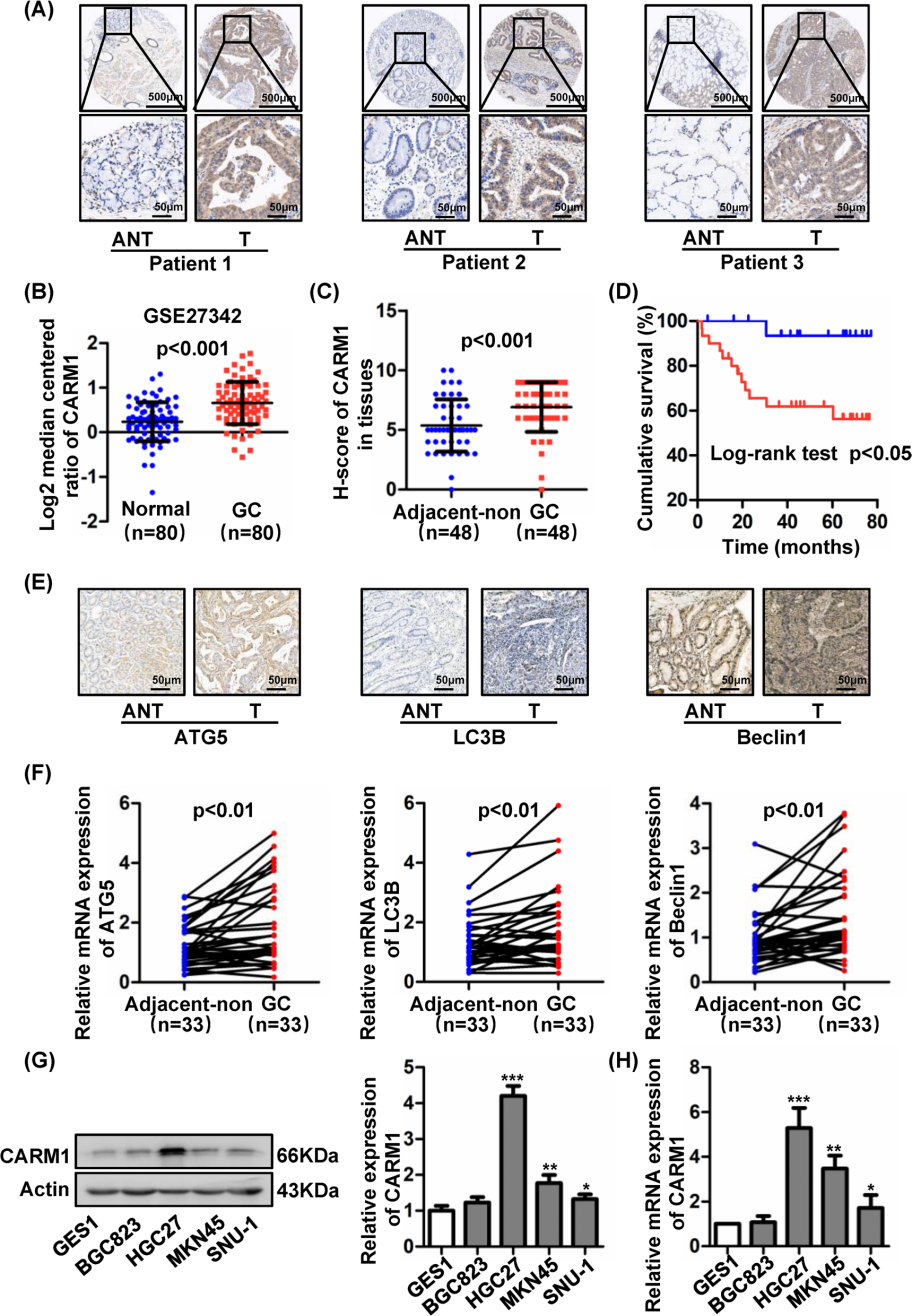
CARM1 and autophagy markers are increased in GC tissues, and higher CARM1 expression predicts worse prognosis. **A** Representative images of immunohistochemistry (IHC) staining of CARM1 in 48 GC tissues and 48 adjacent non-tumor tissues. (Scale bar: above, 500 μm; below, 50 μm). **B** CARM1 mRNA expression was significantly upregulated in GC tissues (n = 80) compared to normal tissues (n = 80) in the TCGA profile (Student’s *t* test, *P* < 0.001). **C** H-score of CARM1 in GC tissues and adjacent non-tumor tissues (n = 48) were evaluated by the staining intensity. (Student’s *t* test, *P* < 0.001). **D** Kaplan–Meier curve depicting the overall survival of GC patients (n = 48) stratified by high and low expression of CARM1 (log-rank test, *P* < 0.05). **E** Representative photographs of IHC staining of the autophagy markers ATG5, LC3B, and Beclin1 in 33 pairs of GC tissues and adjacent non-tumor tissues. (Scale bar: 50 μm). **F** The mRNA expression of ATG5, LC3B, and Beclin1 in 33 pairs of cancer and adjacent tissues was evaluated. **G** Western blot analysis of CARM1 protein expression in human GC cell lines and a gastric mucosal epithelial cell line. β-Actin was used as a loading control. **H** mRNA expression levels of CARM1 in four human GC cell lines and the GES-1 cell line were determined by qRT–PCR. Bars represent the mean ± SD from three independent experiments. *Represents Student's *t* test *P < 0.05, **P < 0.01 and ***P < 0.001

Through analysis of CARM1 expression in TCGA database, we found that CARM1 mRNA expression was upregulated in GC compared to normal tissue samples (80 cases), as shown in Fig. 1B. CARM1 expression was also increased in the GEPIA database (Additional file 1: Fig. S1A). To further investigate the expression of CARM1 in GC tissues, we used GC tissue microarrays containing 48 pairs of samples from patients. IHC staining indicated that CARM1 expression was increased substantially in GC tissues in comparison with adjacent non-tumor tissues (Fig. 1A, C). Furthermore, as shown in Fig. 1D, the overall survival was shorter in patients with higher CARM1 expression, consistent with the results from the KM Plotter database (Additional file 1: Fig. S1B), suggesting that CARM1 was an important prognostic biomarker of GC. Moreover, univariate and multivariate Cox regression analyses demonstrated that CARM1 was an independent risk factor for predicting poor survival (Tables 1, 2). The association of CARM1 level with clinicopathological parameters of GC patients was shown in Additional file 3: Table S1. Because autophagy plays critical roles in tumor progression and CARM1 is known to play a regulatory role in autophagy, we analyzed the expression of autophagy markers in human GC tissues. As detailed in Fig. 1E, ATG5, LC3B and beclin1 protein expression was increased in cancer compared to normal tissues, and the mRNA levels were also higher in cancer according to the analysis of 33 paired clinical samples (Fig. 1F).

**Table 1:** Prognostic factors in gastric cancer patients by univariate analysis

Parameter	n	Cumulative survival rates (%)		Mean survival time (month)	Hazard ratio	95% Confidence interval	*P*- value
		3-year	5-year				
Gender							
Male	30	82.3	76.5	66.128	2.642	0.883–7.905	0.082
Female	18	57.2	57.2	47.510			
Age							
< 60	26	70.5	70.5	58.749	0.911	0.306–2.716	0.867
≥ 60	22	76.5	69.5	61.513			
Location							
Antrum	16	81.3	81.3	64.961			
Cardia fundus	22	70.3	70.3	58.975	1.768	0.44–7.096	0.422
gastric body	7	85.7	85.7	64.611	0.948	0.098–9.122	0.963
Full stomach	3	33.3	0	34.897	6.093	1.216–30.532	0.028
Tumor size							
< 5 cm	29	82.6	82.6	64.143	2.649	0.864–8.124	0.088
≥ 5 cm	19	57.6	48.0	52.100			
Lymph node metastasis							
Negative	20	85.0	85.0	67.608	3.049	0.835–11.132	0.091
Positive	28	63.5	55.6	53.380			
Metastasis							
Negative	44	78.2	73.9	62.904	3.907	1.072–14.234	0.039
Positive	4	25.0	25.0	31.048			
Vessel carcinoma embolus							
Negative	44	73.1	68.8	59.570	0.852	0.111–6.555	0.878
Positive	3	75.0	75.0	61.630			
CARM1 level							
Low	18	93.3	93.3	74.023	8.489	1.103–65.343	0.04
High	30	61.8	56.2	51.956

**Table 2:** Multivariate analysis using the Cox proportional hazards model

Parameter	n	Hazard ratio	95% Confidence interval	*P*-value
Gender				
Male	30	0.887	0.166–4.750	0.888
Female	18			
Age				
< 60	26	1.566	0.336–7.310	0.568
≥ 60	22			
Tumor size				
< 5 cm	29	5.807	0.881–38.294	0.068
≥ 5 cm	19			
Metastasis				
Negative	44	3.106	0.298–32.329	0.343
Positive	4			
CARM1 level				
Low	18	13.107	1.052–63.373	0.046
High	30			

### CARM1 promotes autophagy in GC cells

**Fig. 2:**
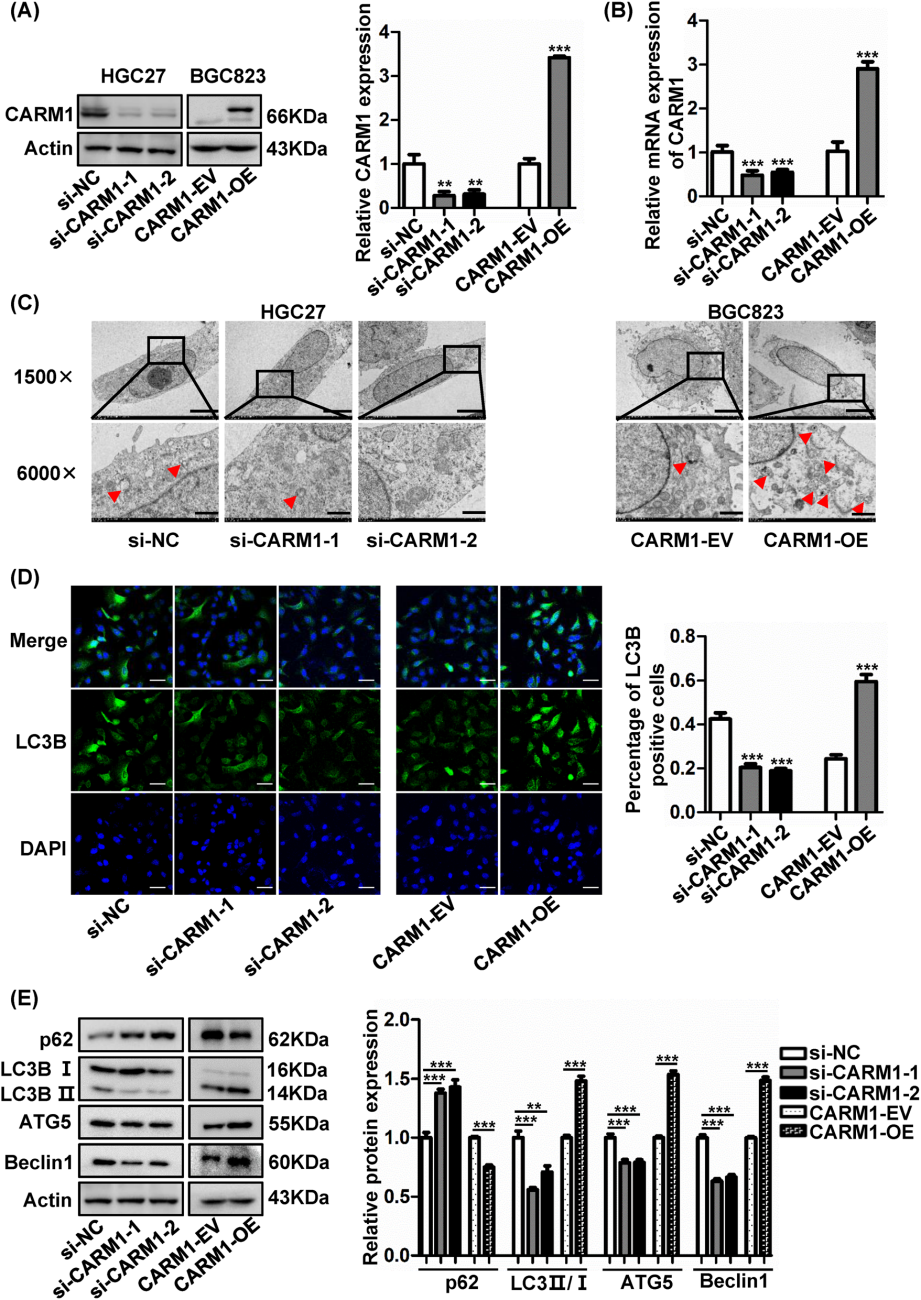
CARM1 enhances autophagy of GC cells. **A** and **B** Knockdown of CARM1 in HGC27 cells and overexpression of CARM1 in BGC823 cells were confirmed by western blotting and qRT–PCR. **C** Representative images of autophagosomes and autolysosomes (indicated by the red arrows) by transmission electron microscopy. The images above are at a lower magnification (scale bar: 5 μm), and the images below are at a higher magnification (scale bar: 1 μm). **D** Representative photographs of immunofluorescence staining showing LC3B-positive cells (scale bar: 50 μm). **E** Protein levels of p62, LC3BII/I, ATG5, and Beclin1 were detected by western blotting using β-Actin as an internal control. (n = 3; error bar, SD). *Represents *P < 0.05, **P < 0.01 and ***P < 0.001

To explore the role of CARM1 in GC cells, we evaluated the expression of CARM1 in 4 GC cell lines and the normal gastric mucosal epithelial cell line GES1. The results revealed that both CARM1 protein and mRNA expression were increased in GC cell lines compared to GES1. CARM1 levels were increased slightly in BGC823 cells but dramatically elevated in HGC27 (Fig. 1G, H). Therefore, we overexpressed CARM1 in BGC823 cells, which has the lowest baseline expression of CARM1, by transfection with lentivirus and downregulated CARM1 in HGC27 cells, which has the highest baseline expression of CARM1, using specific siRNA. The changes in the expression levels of CARM1 in transfected HGC27 and BGC823 cells were confirmed by western blotting and qRT-PCR, as presented in Fig. 2A, B.

Previous studies demonstrated that CARM1 was involved in the regulation of autophagy [11]. Therefore, we morphologically identified autophagosomes and autolysosomes to assess autophagy in GC cells. As shown in Fig. 2C, HGC27 cells with reduced CARM1 expression contained fewer autophagosomes and autolysosomes than control cells, while the number in BGC823 cells transfected with CARM1 overexpression lentivirus increased significantly compared to control cells. Consistent with these results, increases in the number of LC3 puncta, elevated protein expression of LC3 II/I, ATG5 and beclin1, and reduced p62 expression were observed in CARM1-overexpressing cells, which further proved that CARM1 could induce autophagy (Fig. 2D, E).

### Blockage of the autophagy flux induces endoplasmic reticulum stress

**Fig. 3:**
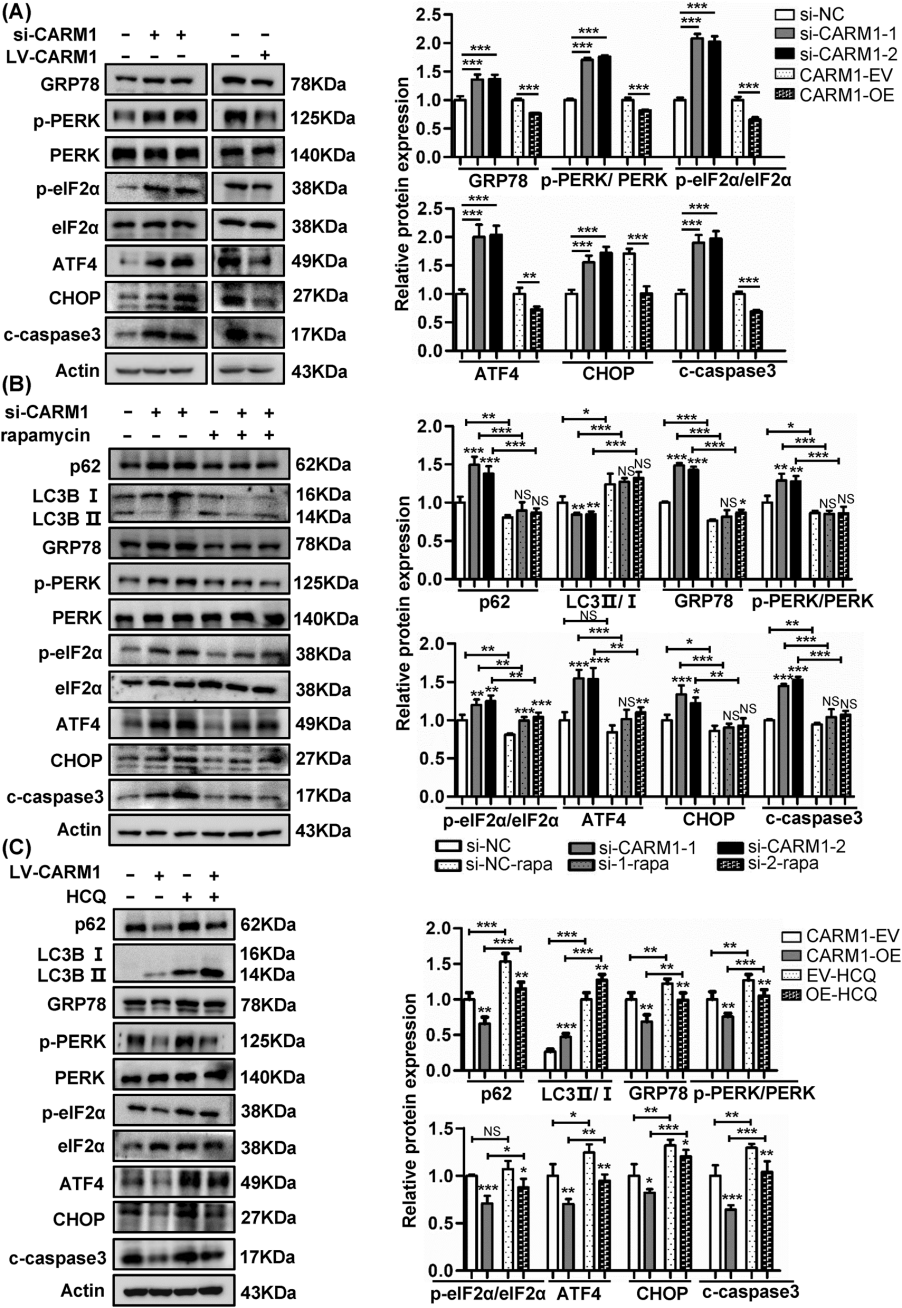
Blockage of autophagy flux induces endoplasmic reticulum (ER) stress. **A** ER stress-related proteins GRP78, p-PERK, PERK, p-eif2α, eif2α, and ATF4 and downstream effector proteins associated with apoptosis were detected by western blotting in CARM1-knockdown HGC27 cells and CARM1-overexpressing BGC823 cells. **B** CARM1-knockdown and control HGC27 cells were treated with rapamycin (50 nM, 24 h) to examine the effect of autophagy induction on ER stress. **C** CARM1-overexpressing and control BGC823 cells were treated with HCQ (25 μM, 24 h) to evaluate the effect of blocking autophagy process on ER stress. β-Actin was applied as loading controls. Each experiment was repeated three times. Data represent mean values ± SD. *Represents *P < 0.05, **P < 0.01 and ***P < 0.001

It has been reported that autophagy and ER stress have complex interactions, and ER stress can induce autophagy through unfolded protein responses (UPRs) [28]. The classical UPR includes three stress transducers, ATF6, IRE1α and PERK [29]. Therefore, we evaluated ER stress-related proteins. IRE1α and ATF6 expression showed no difference in GC cells treated with CARM1 siRNA or overexpression virus (Additional file 1: Fig. S1C). However, the expression of p-PERK, p-eIF2α and ATF4 was elevated in HGC27 cells with CARM1 downregulation but reduced when CARM1 was upregulated, consistent with the change in GRP78, a molecular chaperone of ER that helps proteins fold properly [30] (Fig. 3A). These observations suggested that knockdown of CARM1 triggered ER stress.

On the other hand, a lack of autophagy can in turn promote ER stress, and the inhibition of autophagy causes excess p62 accumulation, which could impair the delivery of polyubiquitinated proteins to induce UPR [31, 32]. Therefore, we utilized the autophagy agonist rapamycin to investigate whether the increased ER stress was caused by the impairment of autophagy. As expected, enhancement of autophagy partially ameliorated ER stress in CARM1-knockdown cells (Fig. 3B). We also used the autophagy inhibitor HCQ to study the changes in ER stress in BGC823 cells. When autophagy was inhibited, the level of ER stress increased (Fig. 3C). In summary, these results showed that impaired autophagy resulted in the accumulation of p62 and thereby induced ER stress.

### CARM1 aggravates the proliferation of GC cells through regulation of autophagy

**Fig. 4:**
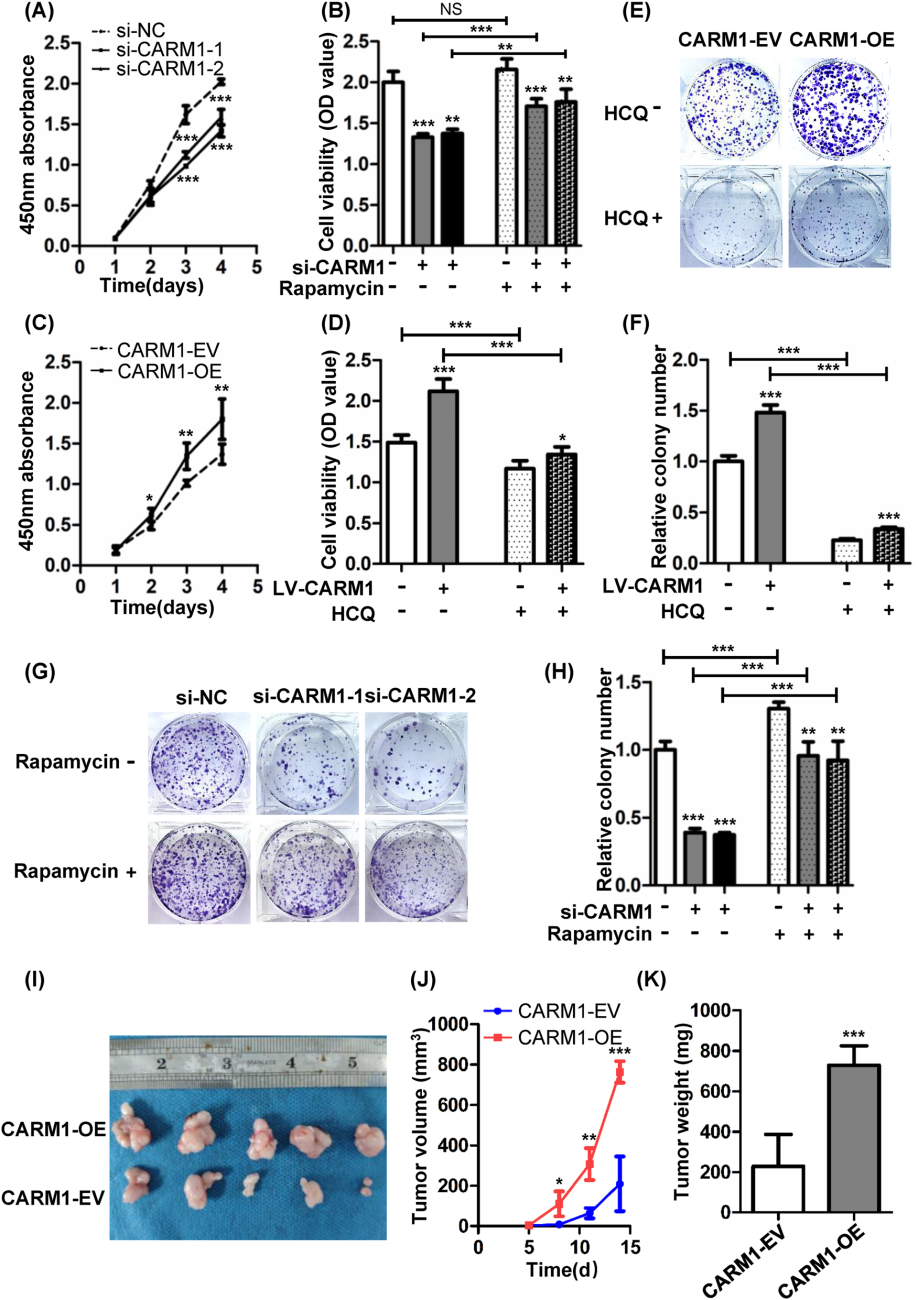
CARM1 promotes the proliferation of GC cells through regulation of autophagy. **A** CCK-8 assay revealed that downregulation of CARM1 significantly suppressed the growth rate in HGC27 cells. **B** CARM1-knockdown and control HGC27 cells were treated with rapamycin (50 nM, 24 h). Cell viability was determined by CCK-8 assay. **C** CCK-8 assay proved that upregulation of CARM1 considerably enhanced cell viability in BGC823 cells. **D** CARM1-overexpressing and control BGC823 cells were treated with HCQ (25 μM, 24 h). Cell viability was determined by CCK-8 assay. **E** and **F** BGC823 cells stably transfected with overexpression or control lentivirus were treated with HCQ (25 μM) 3 days after cells were seeded. Cells were cultured for 10–14 days until visible clones were formed. **G** and **H**, HGC27 cells were transfected with CARM1 siRNA every 5 days to maintain the knockdown effect. Cells were cultured in medium containing rapamycin (50 nM) for 10–14 days until visible clones were formed. Representative images of clones are shown from three independent experiments. **I** Photographs of dissected xenograft tumors showed that the overexpression of CARM1 (top) promoted tumor growth in vivo compared to controls (bottom). **J** Tumor volumes at the indicated time points were evaluated by the formula: tumor volume (mm^3^) = [length (mm) × width (mm)^2^] × π/6. **K** Tumor weights indicated that the xenograft tumors derived from CARM1-overexpressing cells grew faster than those injected with control gastric cancer cells. Data are presented as the mean ± SD. *Represents *P < 0.05, **P < 0.01 and ***P < 0.001

Since autophagy is recognized as a double-edged process in the progression of cancer, we explored the effect of CARM1 and the autophagy induction on GC cell proliferation. CCK-8 analysis revealed that downregulation of CARM1 significantly inhibited cell growth (Fig. 4A); however, when HGC27 cells were treated with the autophagy stimulant rapamycin, the decreased cell viability was partially restored (Fig. 4B). Conversely, BGC823 cells treated with the autophagy suppressant HCQ, which inhibited autophagy flow at the late stage, attenuated the cell viability which was upregulated by CARM1 overexpression (Fig. 4C, D). To further explore the role of autophagy in the progression of GC, we tested another inhibitor, 3-MA, which could inhibit autophagy at the early stage. The inhibitory effect of 3-MA on autophagy was proven by western blotting, as shown in Additional file 2: Fig. S2A. As shown in Additional file 2: Fig. S2B, consistent with the results from the experiment using HCQ, the application of 3-MA also reduced the cell viability which was upregulated by CARM1 overexpression in BGC823 cells. Next, we performed a colony formation assay to verify the role of autophagy induced by CARM1 in cell proliferation. In line with the CCK-8 assay results, upregulation of CARM1 increased the number of clonal colonies, which could be reversed by HCQ (Fig. 4E, F) and 3-MA (Additional file 2: Fig. S2C), while downregulation of CARM1 showed the opposite effect and could be rescued by rapamycin in part (Fig. 4G, H). To further explore the role of CARM1 in tumorigenesis in vivo, we stablished a subcutaneous xenograft model in nude mice. The results showed that overexpression of CARM1 substantially accentuated tumor growth compared to mice injected with CARM1-EV cells (Fig. 4I, J). In keeping with the xenograft tumor volumes, tumors from the nude mice injected with CARM1-overexpressing cells weighed more than the controls (Fig. 4K).

### CARM1 facilitates G1-S cell cycle transition and restrains apoptosis of GC cells by inducing autophagy

**Fig. 5:**
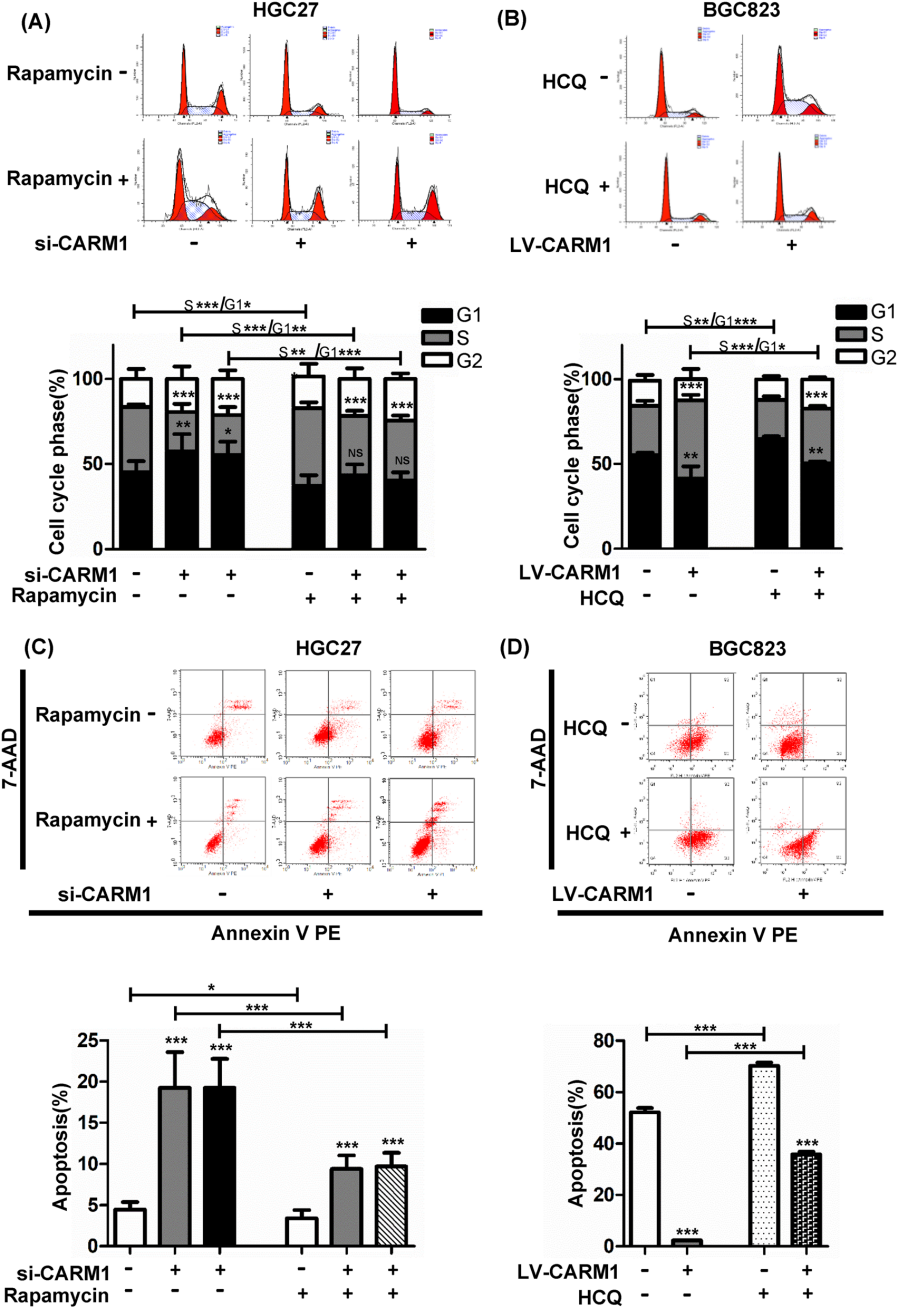
CARM1 potentiates the G1-S transition of the cell cycle and inhibits the apoptosis of GC cells by inducing autophagy. HGC27 cells transfected with si-CARM1 were treated with rapamycin (50 nM, 24 h), and BGC823 cells transduced with overexpression and control lentiviruses were treated with HCQ (25 μM, 24 h) before analysis by flow cytometry. **A** and **B** Distribution of cells in different cell cycle phases determined by flow cytometry is shown for the indicated cells. CARM1 overexpression promoted while CARM1 knockdown attenuated the G1-to-S transition of GC cells. However, the effects could be reversed by the autophagy activator rapamycin and the autophagy inhibitor HCQ. **C** and **D** Representative images showing the percentage of cells undergoing apoptosis from three dependent experiments. Treatment with rapamycin partially reversed the increase in apoptosis in CARM1-knockdown HGC27 cells (left panel), and HCQ recovered the decrease in apoptosis in CARM1-overexpressing cells to some extent (right panel). Bars represent the mean ± SD from three independent experiments. *Represents *P < 0.05, **P < 0.01 and ***P < 0.001

To investigate how CARM1 affects cell proliferation by regulating autophagy, we performed a cell cycle assay by flow cytometry. Figure 5A, B shows that downregulation of CARM1 increased while upregulation decreased the proportion of cells in G0/G1 phase, accompanied by a corresponding reduction (CARM1 downregulation) and increase (CARM1 upregulation) in S phase. Treatment with rapamycin partially recovered the decrease in S phase in CARM1-knockdown HGC27 cells, and HCQ reversed the increase in S phase in CARM1-overexpressing cells to some extent. 3-MA also partially reversed the increase in S phase in CARM1-overexpressing BGC823 cells (Additional file 2: Fig. S2D). These results proved that CARM1 accentuated G1-S transition by regulating autophagy.

Furthermore, apoptosis also plays an important role in the growth of tumor cells. More importantly, as shown in Fig. 3A, B, blockade of autophagy by silencing CARM1 provoked ER stress and subsequently promoted the expression of CHOP and cleaved-caspase3, which are the vital proapoptotic effectors [33]. Therefore, we detected the apoptosis of GC cells under different treatments. As expected, the apoptosis rate was enhanced in CARM1-silenced HGC27 cells, which could be counteracted by rapamycin (Fig. 5C). Opposite effects were confirmed in CARM1-overexpressing cells (Fig. 5D and Additional file 2: Fig. S2E). Therefore, we confirmed that the inhibition of CARM1 lead to the deficient of autophagy flow, resulting in the increase of ER stress-related apoptosis.

### CARM1 inhibitor attenuates the tumor-promoting effect of CARM1

**Fig. 6:**
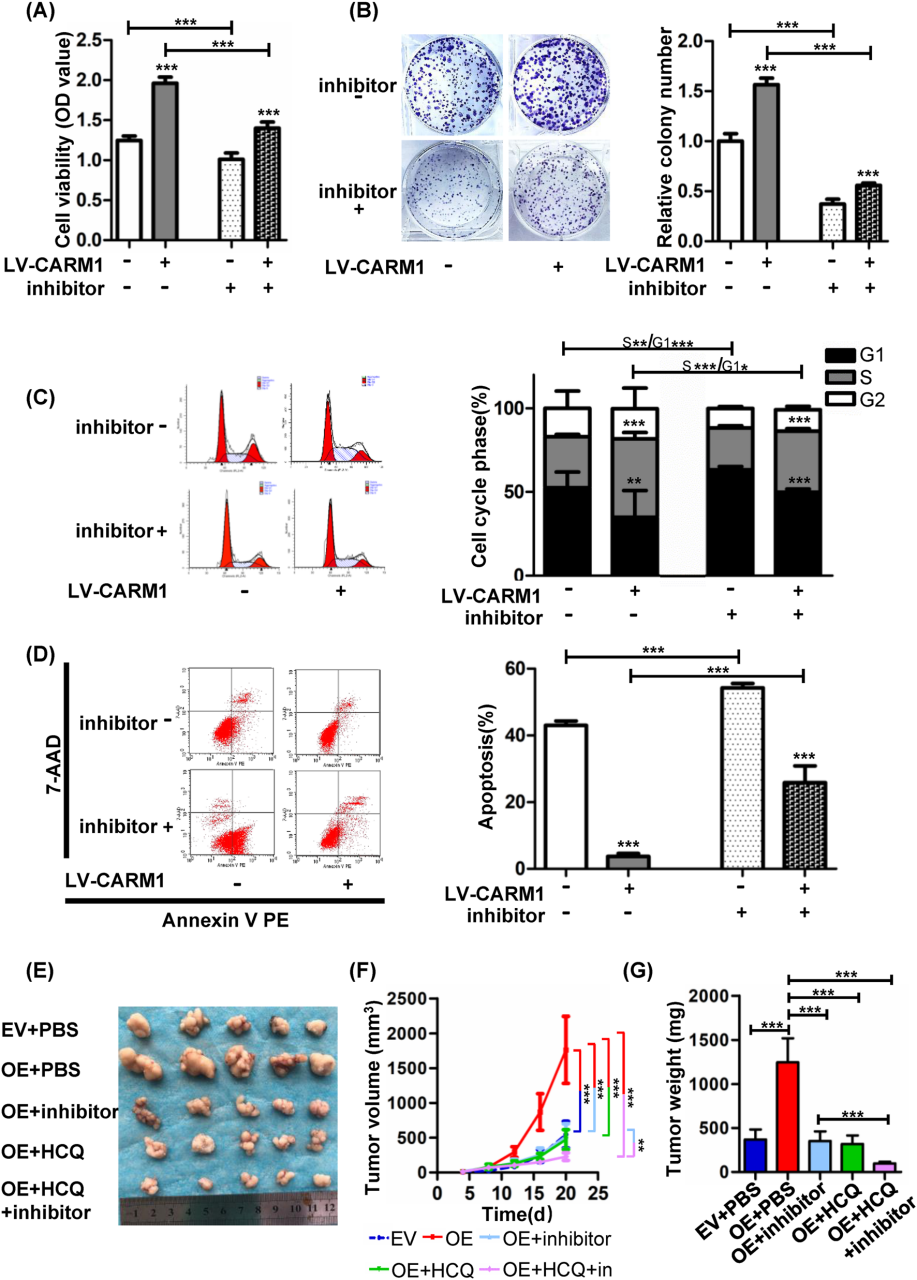
CARM1 inhibitor rescued the tumor-promoting role of CARM1 both in vitro and in vivo. **A** Control and CARM1-overexpressing BGC823 cells were treated with CARM1i (8 μM, 24 h), and cell viability was determined by CCK-8 assay. **B** Control and CARM1-overexpressing BGC823 cells were cultivated in medium containing 8 μM CARM1i for 10–14 days. CARM1i reduced the increase in colony numbers caused by CARM1 overexpression. **C** and **D** Control and CARM1-overexpressing BGC823 cells were treated with CARM1i (8 μM, 24 h). Cell cycle phase distribution and apoptosis in the indicated cells were assessed by flow cytometry. **E**, **F** and **G** BGC823 xenograft models were generated to investigate the therapeutic effect of CARM1i and HCQ on GC in vivo. Nude mice injected with CARM1-overexpressing cells were randomly divided into four groups on day 6 when tumor volumes reached 50 mm^3^. CARM1i injection was performed twice daily at 100 mg/kg i.p. HCQ was administered once daily at 50 mg/kg i.p. The combination group was given two treatments, and the control group was intraperitoneally injected with PBS only. **E** Photograph of subcutaneous tumors dissected from animals in the indicated groups. **F** The tumor volumes were measured every four days and calculated by the formula: tumor volume (mm^3^) = [length (mm) × width (mm)^2^] × π/6. **G** The tumor weights were evaluated. Both CARM1i and HCQ slowed the tumor growth of GC. Furthermore, CARM1i exhibited a synergistic effect in combination with HCQ. Data are demonstrated as the mean ± SD. *Represents *P < 0.05, **P < 0.01 and ***P < 0.001

Given that CARM1 could promote GC tumor growth both in vitro and in vivo, we sought to investigate whether the CARM1 inhibitor (CARM1i) EZM2302 could exert a therapeutic efficacy. As revealed in Fig. 6A, B, CARM1i suppressed the increase in cell viability and clonogenicity induced by CARM1 overexpression. Concordantly, CARM1i also triggered G1 phase cell cycle arrest and significantly reversed the decreased apoptosis rate in CARM1-elevated cells (Fig. 6C, D). Next, we tested the therapeutic effect of CARM1i in mouse xenograft models in vivo*,* and we found that CARM1i had a beneficial therapeutic effect, as demonstrated by the reduced growth rate and eventual reduction in tumor volume, as well as the decreased tumor weight compared to the untreated group injected with CARM1-overexpressing cells (Fig. 6E–G). Interestingly, CARM1i exhibited a synergistic effect when administered in combination with HCQ, suggesting a potential therapeutic strategy for GC (Fig. 6E–G).

### CARM1 activates autophagy by promoting TFE3 nuclear translocation

**Fig. 7:**
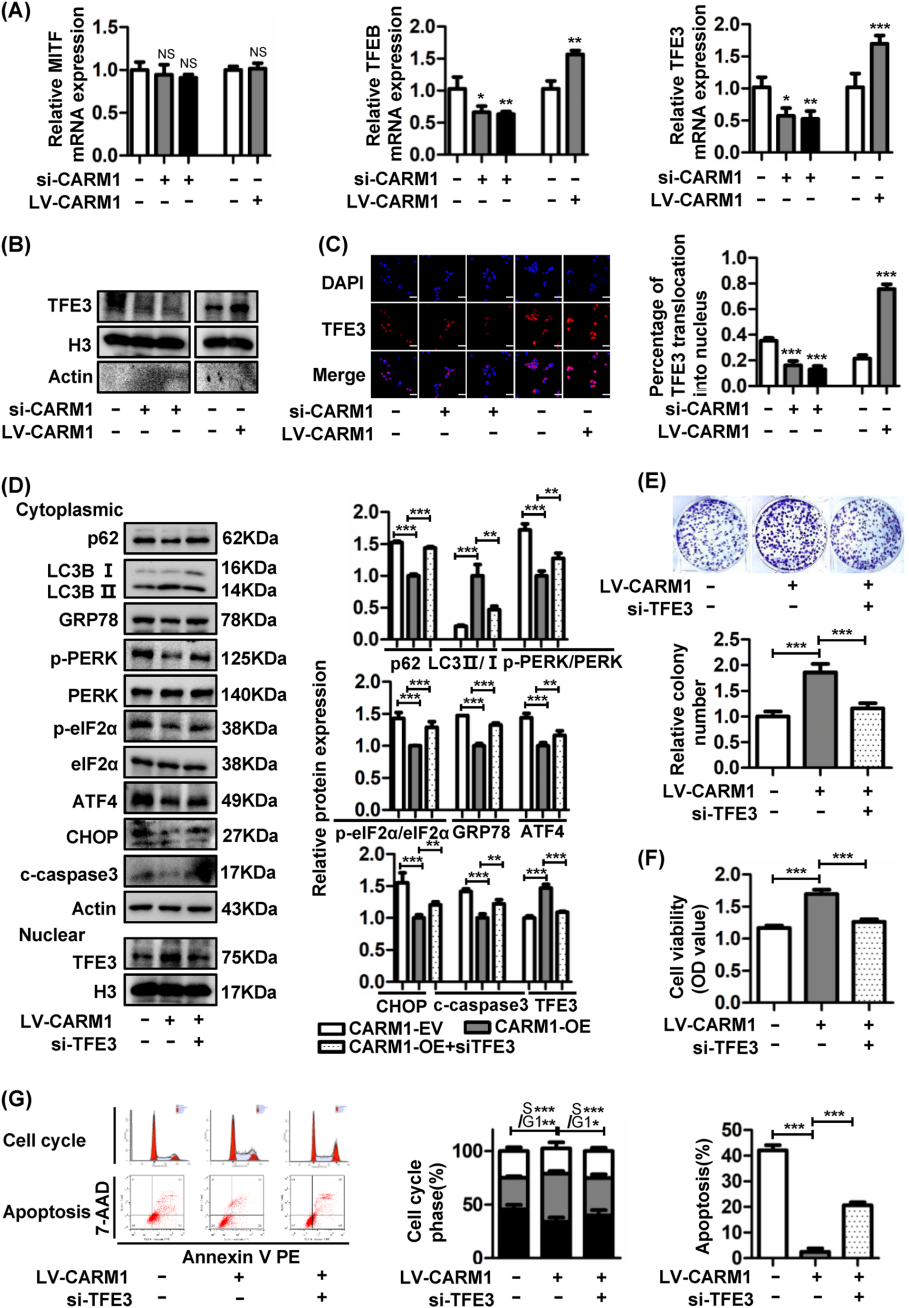
CARM1 activates autophagy by promoting TFE3 nuclear translocation. **A** The mRNA expression of the MiTF/TFE family (TFE3, TFEB, MITF) was detected by qRT–PCR in CARM1-knockdown HGC27 cells and CARM1-overexpressing BGC823 cells. **B** Western blotting was used to assess TFE3 protein levels in the nucleus of CARM1-treated cells, and histone H3 was used as a loading control for nuclear protein. **C** The percentage of TFE3 translocation into the nucleus was evaluated by immunofluorescence (scale bar: 50 μm). **D** Decreased TFE3 expression in CARM1-overexpressing BGC823 cells blocked autophagy flux and subsequently induced ER stress. Autophagy- and ER stress-related protein expression was evaluated in the cytoplasm, and TFE3 expression was examined in the nucleus by western blotting. β-Actin and H3 served as loading controls for cytoplasmic proteins and nuclear proteins, respectively. **E** and **F** Colony formation and CCK-8 assays revealed that TFE3 downregulation reversed the pro-proliferative effect of CARM1 on BGC823 cells. **G** TFE3 knockdown rescued the increased G1-S transition and decreased apoptosis resulting from CARM1 overexpression in BGC823 cells. Bars represent the mean ± SD from three independent experiments. *Represents *P < 0.05, **P < 0.01 and ***P < 0.001

Previous studies have shown that the MiTF/TFE family, including TFE3, TFEB, MITF and TFEC, plays important roles in various physiological processes, especially in lysosomal homeostasis and autophagy regulation [34, 35]. Because TFEC is mainly expressed in bone marrow-derived cells [36], we examined the expression of MITF, TFEB and TFE3 to explore whether the MiTF/TFE family was involved in the regulation of autophagy by CARM1. As shown in Fig. 7A, MITF exhibited no difference, while TFEB and TFE3 were attenuated in CARM1-silenced cells and increased in CARM1-overexpressing cells. CARM1 has been reported to interact with TFEB as its coactivator to promote the transcription of autophagy-related genes [26], and TFE3 and TFEB could share common regulatory networks [37]. Therefore, this study mainly focused on whether CARM1 could promote autophagy by regulating TFE3. Western blotting of nuclear proteins and immunofluorescence staining demonstrated that CARM1 could promote the nuclear translocation of TFE3 (Fig. 7B, C). To illustrate the effect of TFE3 nuclear translocation on autophagy, we knocked down TFE3 expression using siRNA. The TFE3 expression was successfully silenced, and we found that TFE3 knockdown induced a deficiency in autophagy and a subsequent increase in ER stress-mediated apoptotic protein expression (Fig. 7D). Functional experiments further verified that TFE3 knockdown could reverse the increase in cell growth caused by CARM1-induced autophagy (Fig. 7E–G).

### The TFE3 activity is activated through AMPK-mTOR and AMPK-CARM1-TFE3 signaling pathways

**Fig. 8:**
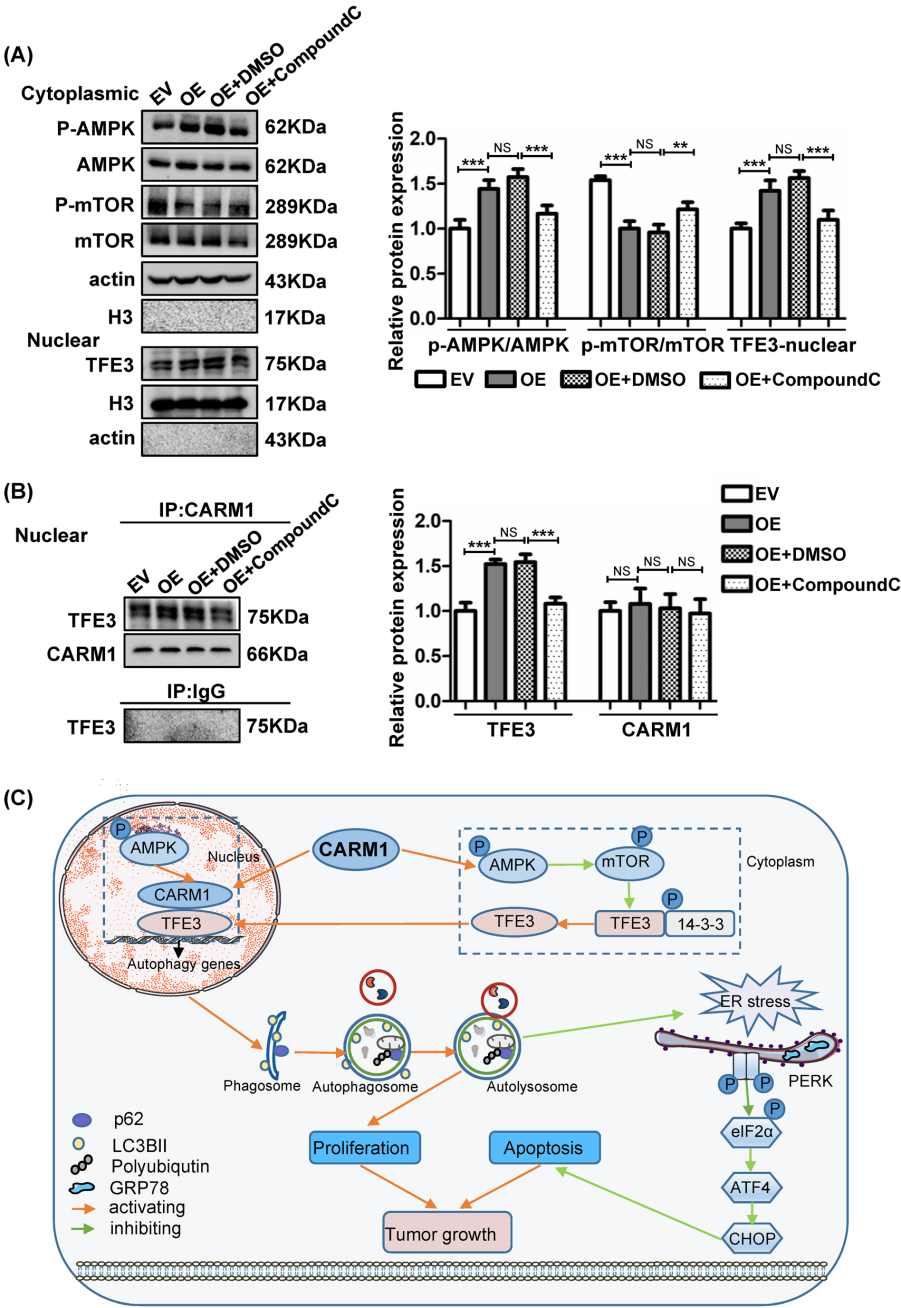
The TFE3 activity is activated via the cytoplasmic AMPK-mTOR and nuclear AMPK-CARM1-TFE3 signaling pathways. **A** Western blotting analysis of the AMPK-mTOR signaling pathway in the cytoplasm and TFE3 expression in the nucleus in the CARM1-EV, CARM1-OE, CARM1-OE + DMSO, and CARM1-OE + Compound C (10 μM, 6 h) cells. **B** CARM1-overexpressing BGC823cells were treated with Compound C (10 μM, 6 h) or the solvent control DMSO. An immunoprecipitation assay using a CARM1 antibody was performed, and the amount of TFE3 protein bound to CARM1 under different experimental conditions was evaluated by western blotting. Values represent the means ± SD. **P < 0.01 and ***P < 0.001. **C** Schematic model for the mechanisms of function of CARM1 in GC. CARM1 potentiates autophagy by facilitating TFE3 nuclear translocation and enhancing its activity by activating cytosolic AMPK-mTOR and nuclear AMPK-CARM1-TFE3 signaling pathways. Then, CARM1 promotes GC cell proliferation and reduces ER stress-induced apoptosis by regulating autophagy

We next tried to clarify the mechanisms by which increased CARM1 promoted the nuclear translocation of TFE3. Previous studies have shown that AMPK-mTOR is an important pathway involved in the regulation of the MiTF/TFE family [34]. Our results demonstrated that in the cytoplasm, p-AMPK/AMPK expression was enhanced while p-mTOR/mTOR was reduced, and that TFE3 nuclear expression was increased in CARM1-overexpressing cells (Fig. 8A). These observations suggested that TFE3 activity was partially regulated by the cytoplasmic AMPK-mTOR signaling pathway. It has been reported that CARM1 can bind to the promoter region of TFEB to enhance transcription [26]. Since TFE3 always shares a regulatory mechanism with TFEB, we conducted an immunoprecipitation assay to explore whether CARM1 could also interact with TFE3. As exhibited in Fig. 8B, the overexpression of CARM1 increased the TFE3 binding level, regardless of different CARM1 expression levels (Fig. 8B). To further investigate the effect of AMPK on TFE3, we applied Compound C, an effective reversible inhibitor of AMPK [38]. As shown in Fig. 8A, B, Compound C suppressed the cytoplasmic AMPK-mTOR pathway and reduced both TFE3 nuclear translocation and binding activity to CARM1.

## Discussion

GC remains one of the deadliest malignancies, and there is a lack of effective early diagnosis and prognostic molecular markers. CARM1 is elevated in various tumors and exhibits a tumor-promoting role mainly as a transcriptional coactivator by methylating histones and non-histones [25, 39]. Paradoxically, CARM1 shows a tumor-inhibiting effect on liver and pancreatic cancers [19, 20]. However, the role of CARM1 and the associated mechanism of pathogenesis in GC have not been studied before. Here, we demonstrated that CARM1 expression was increased by analyzing data in databases and clinical samples of GC and proved for the first time that CARM1 was an independent risk factor for predicting poor survival (Fig. 1A–D). These findings indicate that CARM1 may potentially become a marker for diagnosis and therapeutic decision making for GC.

Recent studies have shown the vital role of CARM1 in autophagy [11]. Autophagy maintains cellular homeostasis through the degradation of long-lived proteins and damaged organelles, and is considered to be a self-defense mechanism against certain stressful conditions [40]. The role of autophagy in tumors is paradoxical depending on the specific environment [41, 42]. On the one hand, autophagy markers are often located in cancer-related DNA regions with frequent mutations or deletions which may be protective mechanisms to inhibit tumor initiation [43], and it has been proven that 5-FU can inhibit GC cell growth and survival by inducing autophagy-related death [44]. On the other hand, autophagy can help tumor cells resist nutritional deficiencies and other cancer-associated conditions to promote tumor progression and chemotherapy resistance [45]. In this study, we demonstrated that CARM1 promoted GC cell proliferation through autophagy-regulated G1-S transition (Figs. 4A–H, 5A, B).

Furthermore, our results showed that defective autophagy process caused by CARM1 inhibition contributed to ER stress-related apoptosis, which also affected tumor growth. There are three main UPR signaling pathways initiated by IRE1α, PERK, and ATF6, respectively, to reduce protein load and enhance protein-folding capacity [46], and we found that the ER stress initiated by CARM1-mediated autophagy was regulated mainly by the PERK pathway (Fig. 3B, C). PERK undergoes dimerization and autophosphorylation, which activates its kinase domain and subsequent phosphorylation of eIF2α. p-eIF2α relieves the unfolded protein burden of ER [47] by inhibiting most protein translation but selectively enhancing ATF4 translation, leading to an increase in downstream protein CHOP expression [48]. CHOP is responsible for the apoptosis of cells with dysfunctional ER [49]. In our study, increased ER stress mediated by impaired autophagy promoted CHOP expression and eventually potentiated apoptosis (Fig. 5C).

Our results demonstrated that CARM1 exerted a significant tumor-promoting role in GC, therefore, we sought to explore the therapeutic effect of targeting CARM1 by utilizing the small molecule compound EZM2302, which is a specific inhibitor of CARM1 and has shown a significant inhibitory effect on multiple myeloma both in vitro and in vivo [21]. In mouse models of AML and diffuse large B-cell lymphoma, CARM1i also exhibited an effective inhibitory effect [50, 51]. Hence, we wished to determine whether CARM1i could also inhibit the progression of GC, a solid tumor. As shown in Fig. 6A–D, CARM1i retarded GC cell viability, induced G1 cell cycle arrest and triggered apoptosis in vitro, and the growth of xenograft tumors in vivo were also suppressed (Fig. 6E–G). Furthermore, the autophagy inhibitor HCQ, which has been proven to possess appreciable antitumor activity in clinical trials [52, 53], demonstrated synergistic antitumor effects with CARM1i, suggesting the combination of CARM1i and HCQ as a potential therapeutic strategy.

Finally, we aimed to clarify how CARM1 regulated autophagy. The MiT/TFE family was reported to exert an essential function in autophagy regulation [54]. We found that TFEB and TFE3 were both increased in CARM1-overexpressing cells (Fig. 7A). As TFEB has been clearly demonstrated to interact with CARM1 to promote autophagy [26], we focused our study on whether CARM1 could induce autophagy by regulating TFE3. TFE3 helps maintain lysosomal homeostasis and promotes autolysosome formation by binding to the CLEAR elements of lysosome- and autophagy-relevant genes [55]. The results showed that CARM1 promoted TFE3 nuclear translocation to activate autophagy, and silencing TFE3 reversed the increased cell proliferation and decreased apoptosis induced by CARM1-mediated autophagy. Then, we further explored the mechanisms by which increased CARM1 promoted the nuclear translocation of TFE3. AMPK is a responder to starvation and low energy states to maintain intracellular energy homeostasis. Previous studies have shown that AMPK is often activated during the development of GC, demonstrating a pro-tumor effect [56, 57]. AMPK is considered to be a key signaling pathway regulating the activity of the MiT/TFE transcription factor family [34]. It has been reported that TFE3 nuclear translocation is closely related to the activation of the AMPK-mTOR signaling pathway in the cytoplasm. As reported, MTOR complex 1 (MTORC1) is the most important regulator of TFE3 which directly phosphorylates TFE3 serine residue 321 and causes TFE3 cytoplasmic retention [58]. MTORC1 activity is negatively regulated by AMPK through direct and indirect phosphorylation under energy deficiency [59, 60]. Moreover, activated AMPK could directly phosphorylate TFE3 serine residues and regulate TFE3 transcriptional activity. Therefore, we investigated the cytoplasmic AMPK-mTOR pathway and found that the activated AMPK-mTOR pathway was responsible for the nuclear translocation of TFE3 (Fig. 8A). Furthermore, AMPK also regulates the MiT/TFE family through nuclear AMPK-CARM1 signaling pathway. When activated in the nucleus, AMPK phosphorylates FOXO3a and transcriptionally inhibits SKP2, thereby reducing the ubiquitination of CARM1 and increasing the protein level of CARM1 in the nucleus [26]. CARM1 was reported to coactivate TFEB in the nucleus, and TFEB and TFE3 always shared regulatory networks. We then explored the interaction of CARM1 and TFE3 in the nucleus and proved that CARM1 could also regulate autophagy through the nuclear AMPK-CARM1-TFE3 signaling pathway (Fig. 8B). Furthermore, it has been reported that CARM1 can regulate the activation of AMPK in skeletal muscle during denervation-induced plasticity. CARM1 interacts with AMPK and causes the methylation of AMPK, resulting in AMPK activation [61]. This result is consistent with the increased AMPK activation in CARM1-overexpressing cells in our study. These results suggest that there may be a regulatory loop between CARM1 and AMPK which forms positive feedback to promote tumor development. And the detailed mechanism and structure–function relationship are needed to be further explored.

In conclusion, the present study first demonstrated that CARM1 was upregulated in clinical GC tissues and cell lines, and that higher CARM1 expression was related to an unfavorable prognosis. More importantly, our study proved for the first time that CARM1i treatment significantly inhibited GC tumor growth both in vivo and in vitro and synergized with autophagy inhibitors, suggesting a promising therapeutic strategy. CARM1 promoted GC cell proliferation, accelerated G1-S transition and reduced ER stress-induced apoptosis by regulating autophagy. Mechanistically, CARM1 potentiated autophagy by facilitating TFE3 nuclear translocation and enhancing its activity through the activation of cytosolic AMPK-mTOR and nuclear AMPK-CARM1-TFE3 signaling pathways.

## Supplementary Information

**Additional file 1: Figure S1. **A Gene expression profile across all tumor samples and paired normal tissues from the GEPIA database. CARM1 expression was increased in gastric cancer tissues compared to normal tissues (indicated by the red box). B, Data from the KM Plotter database suggested that overall survival was shorter in GC patients with higher CARM1 expression. C, The IRE1α and ATF6α protein expression levels were evaluated by western blotting. Bars represent the mean ± SD from three independent experiments.

**Additional file 2: Figure S2.** Autophagy inhibitor 3-MA rescued the tumor-promoting role of CARM1. A Protein levels of p62 and LC3BII/I were determined by western blotting using β-Actin as an internal control. B Control and CARM1-overexpressing BGC823 cells were treated with 3-MA (5 mM, 24 h), and cell viability was examined by CCK-8 assay. C BGC823 cells stably transfected with overexpression or control lentivirus were treated with3-MA (5 mM) 4 days after cells were seeded. Cells were cultured for 10–14 days until visible clones were formed. The colony formation assay revealed that 3-MA reversed the pro-proliferative effect caused by CARM1 overexpression. D and E Control and CARM1-overexpressing BGC823 cells were treated with 3-MA (5 mM, 24 h). D Distribution in different cell cycle phases was analyzed for the indicated cells by flow cytometry. E The percentage of cells undergoing apoptosis was determined by flow cytometry. Data are expressed as the mean ± SD. *Represents *P < 0.05, **P < 0.01 and ***P < 0.001.

**Additional file 3: Table S1.** Association of CARM1 level with clinicopathological parameters of patients with gastric cancer.

**Additional file 4: Table S2.** Sequences of primers and targets for siRNA.

**Additional file 5: Table S3.** Antibodies and regents used in experiments.

## Data Availability

The data and materials supporting the findings of the research are available from the corresponding author.

## Abbreviations

GC: Gastric cancer
CARM1: Coactivator-associated arginine methyltransferase-1
IHC: Immunohistochemistry
DMEM: Dulbecco’s modified Eagle’s medium
ER stress: Endoplasmic reticulum stress
MITF: Melanocyte inducing transcription factor
TFE3: Transcription factor binding to IGHM enhancer 3
TFEB: Transcription factor EB
