# Supplementary figures and images for: CARM1 promotes gastric cancer progression by regulating TFE3 mediated autophagy enhancement through the cytoplasmic AMPK-mTOR and nuclear AMPK-CARM1-TFE3 signaling pathways
Source: Cancer Cell Int. 2022 Mar 4;22:102. doi: 10.1186/s12935-022-02522-0 (PMC8895580; doi:10.1186/s12935-022-02522-0)

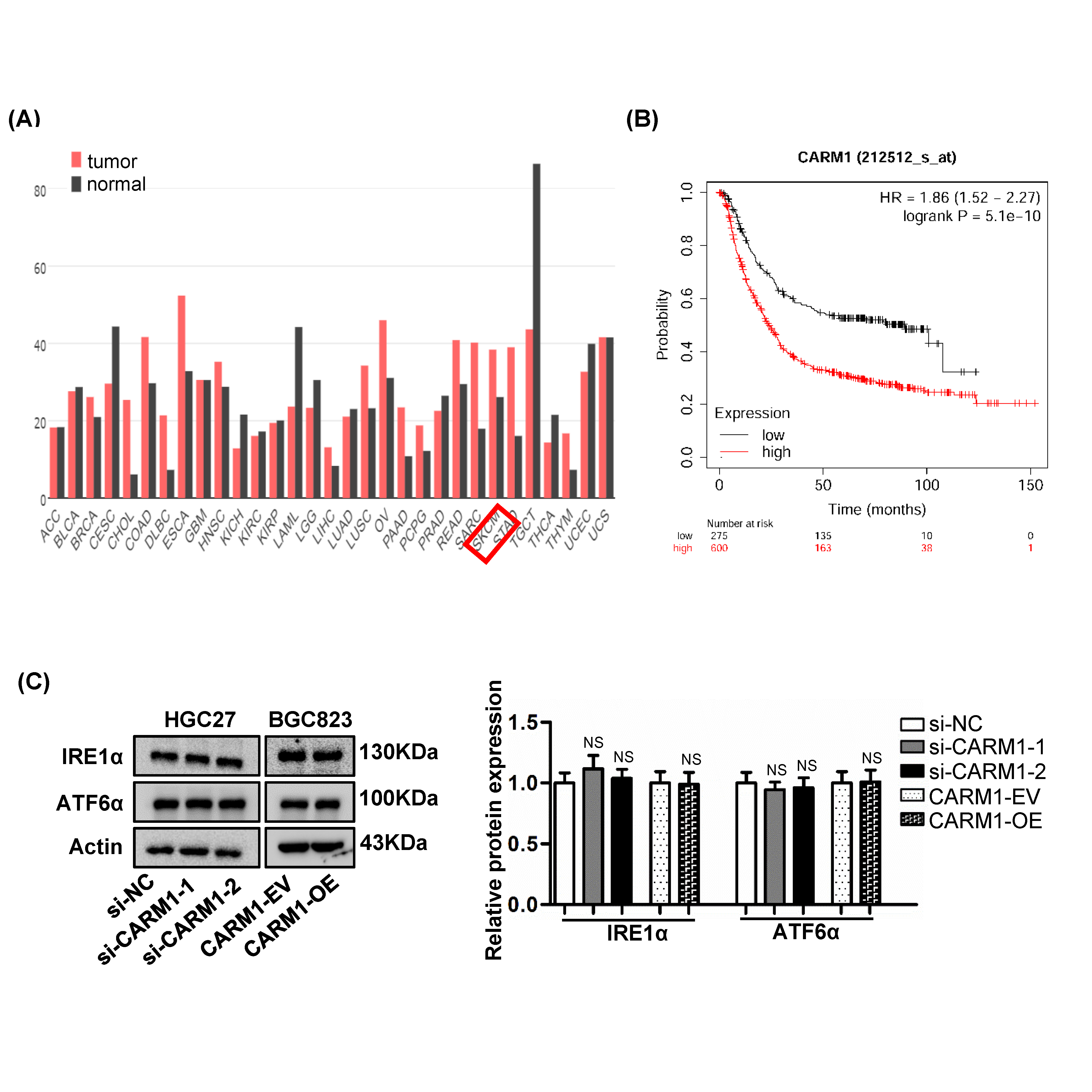

Supplement: Supplementary file 1 — Additional file 1: Figure S1. A Gene expression profile across all tumor samples and paired normal tissues from the GEPIA database. CARM1 expression was increased in gastric cancer tissues compared to normal tissues (indicated by the red box). B, Data from the KM Plotter database suggested that overall survival was shorter in GC patients with higher CARM1 expression. C, The IRE1α and ATF6α protein expression levels were evaluated by western blotting. Bars represent the mean ± SD from three independent experiments. [file 12935_2022_2522_MOESM1_ESM.tiff]

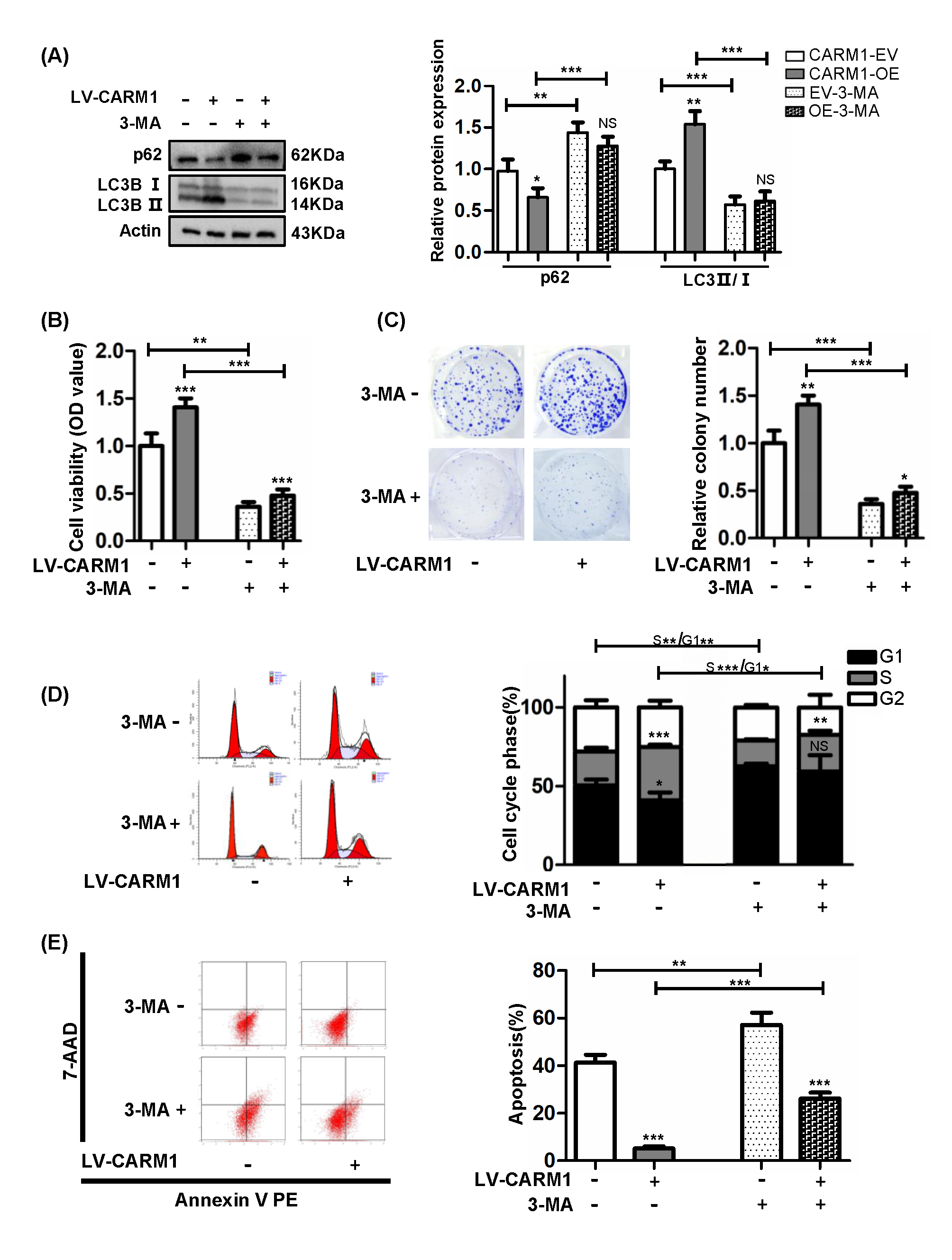

Supplement: Supplementary file 2 — Additional file 2: Figure S2. Autophagy inhibitor 3-MA rescued the tumor-promoting role of CARM1. A Protein levels of p62 and LC3BII/I were determined by western blotting using β-Actin as an internal control. B Control and CARM1-overexpressing BGC823 cells were treated with 3-MA (5 mM, 24 h), and cell viability was examined by CCK-8 assay. C BGC823 cells stably transfected with overexpression or control lentivirus were treated with3-MA (5 mM) 4 days after cells were seeded. Cells were cultured for 10–14 days until visible clones were formed. The colony formation assay revealed that 3-MA reversed the pro-proliferative effect caused by CARM1 overexpression. D and E Control and CARM1-overexpressing BGC823 cells were treated with 3-MA (5 mM, 24 h). D Distribution in different cell cycle phases was analyzed for the indicated cells by flow cytometry. E The percentage of cells undergoing apoptosis was determined by flow cytometry. Data are expressed as the mean ± SD. *Represents *P < 0.05, **P < 0.01 and ***P < 0.001. [file 12935_2022_2522_MOESM2_ESM.tiff]
