# Supplementary material for: CARM1 promotes gastric cancer progression by regulating TFE3 mediated autophagy enhancement through the cytoplasmic AMPK-mTOR and nuclear AMPK-CARM1-TFE3 signaling pathways
Source: Cancer Cell Int. 2022 Mar 4;22:102. doi: 10.1186/s12935-022-02522-0 (PMC8895580; doi:10.1186/s12935-022-02522-0)
Supplement: Supplementary file 3 — Additional file 3: Table S1. Association of CARM1 level with clinicopathological parameters of patients with gastric cancer. [file 12935_2022_2522_MOESM3_ESM.docx]

**Table S1** Association of CARM1 level with clinicopathological parameters of patients with gastric cancer

| Parameter | n | CARM1 level | | *p*-value |
| --- | --- | --- | --- | --- |
|  |  | **Low** | **High** |  |
| Gender |  |  |  |  |
| Male | 30 | 14 | 16 |  |
| Female | 18 | 4 | 14 | 0.090 |
| Age |  |  |  |  |
| <60 | 26 | 9 | 17 |  |
| ≥60 | 22 | 9 | 13 | 0.654 |
| Location |  |  |  |  |
| Antrum | 16 | 6 | 10 |  |
| Cardia fundus | 22 | 10 | 12 |  |
| gastric body | 7 | 2 | 5 |  |
| Full stomach | 3 | 0 | 3 | 0.572 |
| Tumor size |  |  |  |  |
| <5 cm | 29 | 11 | 18 |  |
| ≥5 cm | 19 | 7 | 12 | 0.939 |
| Lymph node metastasis |  |  |  |  |
| Negative | 20 | 8 | 12 |  |
| Positive | 28 | 10 | 18 | 0.762 |
| Metastasis |  |  |  |  |
| Negative | 44 | 18 | 26 |  |
| Positive | 4 | 0 | 4 | 0.282 |
| Vessel carcinoma embolus | |  |  |  |
| Negative | 44 | 17 | 27 |  |
| Positive | 4 | 1 | 3 | 0.516 |
