# Supplementary material for: CARM1 promotes gastric cancer progression by regulating TFE3 mediated autophagy enhancement through the cytoplasmic AMPK-mTOR and nuclear AMPK-CARM1-TFE3 signaling pathways
Source: Cancer Cell Int. 2022 Mar 4;22:102. doi: 10.1186/s12935-022-02522-0 (PMC8895580; doi:10.1186/s12935-022-02522-0)
Supplement: Supplementary file 4 — Additional file 4: Table S2. Sequences of primers and targets for siRNA. [file 12935_2022_2522_MOESM4_ESM.docx]

**Table S2** Sequences of primers and targets for siRNA

| **Gene** | **Sequence** | **Description** |
| --- | --- | --- |
| CARM1 | TTCCAGTCACCACTGTTCGCCA | Forward primer |
|  | CCAGGAGGTTACTGGACTTGGA | Reverse primer |
| ATG5 | GCAGATGGACAGTTGCACACAC | Forward primer |
|  | GAGGTGTTTCCAACATTGGCTCA | Reverse primer |
| LC3B | GAGAAGCAGCTTCCTGTTCTGG | Forward primer |
|  | GTGTCCGTTCACCAACAGGAAG | Reverse primer |
| Beclin1 | CTGGACACTCAGCTCAACGTCA | Forward primer |
|  | CTCTAGTGCCAGCTCCTTTAGC | Reverse primer |
| MITF | CATTCTCAAGGCCTCTGTGGACTA | Forward primer |
|  | GTGCCGAGGTTGTTGGTAAAGGTG | Reverse primer |
| TFEB | AAGGAGCGGCAGAAGAAAGA | Forward primer |
|  | CCAACTCCTTGATGCGGTCA | Reverse primer |
| TFE3 | CAGCTGCTCAGCCTGAACTC | Forward primer |
|  | CTTGAGCGAAGGGGTAAGGG | Reverse primer |
| β-Actin | GGTCATCACCATTGGCAA | Forward primer |
|  | GAGTTGAAGGTAGTTTCGTGGA | Reverse primer |
| CARM1 | GGAUAGAAAUCCCAUUCAATT | Si-CARM1-1 sense |
|  | UUGAAUGGGAUUUCUAUCCTT | Si-CARM1-1 antisense |
| CARM1 | GGACAAGAUCGUUCUUGAUTT | Si-CARM1-2 sense |
|  | AUCAAGAACGAUCUUGUCCTT | Si-CARM1-2 antisense |
| TFE3 | GCUCCGAAUUCAGGAACUATT | Si-TFE3 sense |
|  | UAGUUCCUGAAUUCGGAGCTT | Si-TFE3 antisense |
