# Supplementary material for: CARM1 promotes gastric cancer progression by regulating TFE3 mediated autophagy enhancement through the cytoplasmic AMPK-mTOR and nuclear AMPK-CARM1-TFE3 signaling pathways
Source: Cancer Cell Int. 2022 Mar 4;22:102. doi: 10.1186/s12935-022-02522-0 (PMC8895580; doi:10.1186/s12935-022-02522-0)
Supplement: Supplementary file 5 — Additional file 5: Table S3. Antibodies and regents used in experiments. [file 12935_2022_2522_MOESM5_ESM.docx]

**Table S3** Antibodies and regents used in experiments

| **Antibody/ Regent** | **Product code** | **Application** |
| --- | --- | --- |
| CARM1 | A300-421A; Bethyl | IHC 1:50 |
| ATG5 | ab108327; Abcam | IHC 1:100 |
| LC3B | ab232940; Abcam | IHC 1:100 |
| Beclin1 | ab210498; Abcam | IHC 1:100 |
| CARM1 | A300-421A; Bethyl | Western blotting 1:1000 |
| ATG5 | ab108327; Abcam | Western blotting 1:1000 |
| LC3B | ab192890; Abcam | Western blotting 1:2000 |
| Beclin1 | ab210498; Abcam | Western blotting 1:1000 |
| P62 | ab109012; Abcam | Western blotting 1:2000 |
| GRP78 | ab108615; Abcam | Western blotting 1:2000 |
| p-PERK | 12379; Signal way Antibody | Western blotting 1:1000 |
| PERK | ab229912; Abcam | Western blotting 1:1000 |
| p-eIF2α | 3398; CST | Western blotting 1:1000 |
| eIF2α | 5324; CST | Western blotting 1:1000 |
| ATF4 | 11815; CST | Western blotting 1:1000 |
| CHOP | 2895; CST | Western blotting 1:500 |
| c-caspase3 | 9664; CST | Western blotting 1:500 |
| TFE3 | 14480-1-AP; Proteintech | Western blotting 1:500 |
| p-AMPK | 2537; CST | Western blotting 1:1000 |
| AMPK | 5832; CST | Western blotting 1:1000 |
| p-mTOR | ab109268; Abcam | Western blotting 1:2000 |
| mTOR | ab32028; Abcam | Western blotting 1:1000 |
| β-Actin | NC011; Zhuangzhi | Western blotting 1:1000 |
| Histone H3 | 4499; CST | Western blotting 1:1000 |
| LC3B | ab192890; Abcam | IF 1:50 |
| TFE3 | HPA023881;MilliporeSigma | IF 1:50 |
| CARM1 | 4438; CST | IP 1:50 |
| Rapamycin | HY-10219; MCE |  |
| Hydroxychloroquine Sulfate  3-Methyladenine  Compound C | GC12544; Glpbio  GC10710; Glpbio  GC17243; Glpbio |  |
| EZM2302 | GC19149; Glpbio |  |
